# Treatment of Active Mucositis With Caphosol (Calcium Phosphate): A Retrospective Case-Series

**DOI:** 10.4021/wjon683e

**Published:** 2013-07-15

**Authors:** Jawaid Younus, Lynda Kligman, M. A. Jawaid, Ally Dhalla

**Affiliations:** a Department of Medical Oncology, London Regional Cancer Program, London, ON N6A 4L6, Canada; b Department of Nursing, London Regional Cancer Program, Canada; c Department of Social Sciences, University of Western Ontario, Canada; d Department of Pharmacy, London Regional Cancer Program, London, ON N6A 4L6, Canada

**Keywords:** Mucositis, Chemotherapy, Caphosol, Calcium phosphate

## Abstract

**Background:**

Mucositis is a common side effect due to chemo and/radiation therapy. Caphosol has been a proven preventive strategy against mucositis in randomized clinical trials. However, its efficacy to treat active mucositis in patients treated for solid tumors with chemotherapy is unknown. The objective was to evaluate the efficacy of Caphosol to treat mucositis by comparing the grade of mucositis before and after treatment and documenting the duration of treatment.

**Methods:**

A retrospective review was conducted on consecutive adult patients at London Regional Cancer Program (LRCP) who developed chemotherapy-induced oral mucositis and were then treated with Caphosol. This study was approved by ethics committee at University of Western Ontario.

**Results:**

A total of 21 patients, two males (one with cancer esophagus and another with lung cancer) and 19 females (all with breast cancer), with a median age of 59 years were evaluated. Grade 3 mucositis was present in 4 patients who completely resolved with Caphosol in an average of 4 days of treatment, without needing any hospitalization. Fifteen patients with grade 2 mucositis reverted back to grade 0 by using Caphosol for an average of 3.5 days. One patient with no effect had grade 1 mucositis dating prior to treatment with chemotherapy and remained as such. Another patient with no initial improvement had oral candidiasis and once treated with Fluconozole and Caphosol had a complete resolution. No obvious side effects were reported by patients related to the use of Caphosol.

**Conclusion:**

Our case series, for the first time, shows that Caphosol may be used as a potentially effective treatment in patients with solid tumor, who develop chemotherapy-induced mucositis.

## Introduction

Oral mucositis is a common side effect induced by chemotherapy and or radiation therapy [1, 2]. Although the incidence varies with the chemotherapy agents and the use of overlapping radiation therapy, it is estimated that oral mucositis may be present in about 35% of such patients [1, 2]. The incidence is likely higher [3] with targeted therapies against epidermal growth factor receptor (like Gefitinib and Erlotinib) and with mTOR inhibitors (for example: Everolimus). The mucosal injury process may require several steps but results in mild to moderate discomfort in the mouth with erythema and/ulceration (grade 1 and 2 respectively). The progression causes inability to eat and may require intra-venous fluid/enteral support usually in the hospital setting (grade 3 and 4 severity respectively).

The treatment of mucositis has been largely symptomatic with the use of routine oral care, the use of soft toothbrush, soft and less irritating foods and analgesia. The present literature [4] has no support for the use of benzydamine HCl, sucralfate, tetrachlorodecaoxide, chlorhexidine and “magic mouthwash” (lidocaine solution, diphenhydramine hydrochloride and aluminum hydroxide suspension).

Neutral supersaturated calcium phosphate rinse (Caphosol) has been found to be an effective preventive strategy against mucositis [5, 6]. Caphosol reduced the duration of days with mucositis as well as the pain severity along with the reduction in the use of narcotic analgesics among the treated patients. Although the exact mechanism is unknown, Caphosol may help mucositis by altering oral pH and providing calcium and phosphate ions to the local tissues for faster wound healing [5]. There is presently no data regarding the use or efficacy of Caphosol against the developed mucositis inpatients with solid tumors. Here we present a case series of 21 consecutive patients with chemotherapy-induced mucositis treated with Caphosol.

## Study Design/Method

We conducted a retrospective chart review on adult oncology patients, treated at London Regional Cancer Program, London, ON, who developed chemotherapy-induced mucositis and were treated with Caphosol. These patients were not treated with radiation therapy either prior to concomitant with chemotherapy. No other mouth rinse or mouth wash was used in the study patients. This study was approved by ethics committee at University of Western Ontario. Caphosol was provided as a compassionate supply by Suparna Pharma, Canada. These patients who were prescribed and provided with Caphosol from this compassionate supply program between June and November of 2012 were tracked through the LRCP pharmacy. Their age, diagnosis, chemotherapy regimen, grade of mucositis before and after the use of Caphosol, side effects with the use of Caphosol, frequency of Caphosol use per day and total number of days taken to achieve the best response were recorded from their electronic and paper charts. These patients were instructed to combine the two ampoules (calcium and phosphate) and use it as swish and spit in two quick sessions for each dose, four times daily till resolution of symptoms. This was a pilot observational study and therefore no statistical computations were considered for the design or results.

## Results

A total of 21 patients were evaluated. The median age was 59 years. Majority (19) of patients had the diagnosis of breast cancer, while one patient had cancer of esophagus and another patient had non-small cell lung cancer. Both of these patients were males. The chemotherapy regimens for these patients as well as other parameters are listed in Table 1. The most severe grade of mucositis prior to Caphosol treatment was 3, present in 4 patients. All four patients experienced complete resolution of mucositis after treatment with Caphosol. Among these four patients, the duration of Caphosol treatment was three days for two patients and 5 days for other two patients, with average of 4 days. Two patients used it four times daily while the other two used it twice or three times. These patients were able to avoid hospitalization. Grade 2 mucositis was present in 15 patients prior to the treatment. This was reverted back to grade 0 by using Caphosol for an average of 3.5 days (range: 2 - 7 days). One patient who had grade 1 mucositis had no improvement with Caphosol therapy. It was later confirmed that this patient had her symptoms dating back to several months prior to starting chemotherapy. Another patient who presented with grade 2 mucositis experienced no initial improvement with Caphosol. On a subsequent visit she was found to have oral candidiasis. Fluconozole was added to the Caphosol therapy and the patient had a complete resolution within 5 days. The most common frequency of Caphosol use was recorded as 4 times per day (range: 2 - 4). No obvious side effects were reported by patients with the use of Caphosol.

**Table 1:** The Chemotherapy Regimens and Other Parameters

Age (Yrs)	Diagnosis	Chemotherapy	Worst Grade Initial	Grade - Post Treatment	Duration of Treatment (Days)	Frequency X/Day
1. 72	Non-Small Cell Lung Ca	Carboplatin + Vinblastine	2	0	1	4
2. 54	Breast Ca	TC	3	0	5	4
3. 50	Breast Ca	AC x 4 Doc x 4 Herceptin	2	0	2	4
4. 57	Breast Ca	FEC-D	2	0	2	2
5. 68	Breast Ca	AC/T Dose dense	2	0	5	4
6. 76	Breast Ca	FEC-D	3	0	3	2
7. 47	Breast Ca	TC	2	0	2	4
8. 67	Breast Ca	CMF	1	1	5	4
9. 51	Breast Ca	AC/T Dose dense Herceptin	2	2	7	4
10. 50	Breast Ca	AC/T Dose dense	2	0	2	2
11. 67	Esophageal Ca	ECF	3	0	5	4
12. 59	Breast Ca	Weekly Doc	2	0	3	3
13. 45	Breast Ca	Capecitibine	3	0	7	4
14. 70	Breast Ca	FEC-D	2	1	3	2
15. 53	Breast Ca	FEC-D	2	0	6	3
16. 64	Breast Ca	FEC-D	2	0	7	4
17. 60	Breast Ca	Cis + Gem	2	0	5	4
18. 40	Breast Ca	FEC-D	2	0	3	4
19. 59	Breast Ca	FEC-D	2	0	5	4
20. 63	Breast Ca	TC	2	0	3	4
21. 57	Breast Ca	FEC-D	2	0	4	4

TC: Taxotere and Cyclophosphamide, AC: Adriamycin and Cyclophosphamide, Doc: Docetaxel (Taxotere), FEC-D: 5 Flourouracil, Epirubicin and Cyclophosphamide-Taxotere, AC/T: Adriamycin, Cyclophosphamide and Paclitaxel, CMF: Cyclophosphamide, Methotrexate and 5 Flourouracil, ECF: Epirubicin, Cisplatin and 5 Flourouracil, Cis + Gem: Cisplatin and Gemcitibine.

## Discussion

Oral mucositis continues to be a challenging symptom to the treating physicians as very few therapeutic options are currently available. The most recent update by the mucositis study group from multi-national association of supportive care in cancer (MASCC) does not list any active treatment in patients who have developed chemotherapy-induced mucositis [7]. We treated 21 patients with Caphosol, who presented with variable grades of mucositis induced by chemotherapy. Almost all patients in our case series experienced complete resolution of their symptoms with oral mucositis. The duration of Caphosol therapy was only 4 - 5 days with no reported side effects.

The efficacy of Caphosol (calcium-phosphate solution) as a preventive strategy against mucositis is well established through prospective clinical trials [5, 6]. As a preventive strategy, Caphosol treatment duration of few weeks may be well justified with the high proportion of patients developing significant mucositis, particularly among patients with head and neck cancer or in patients undergoing high dose chemo-radiation therapy for bone marrow transplant. The efficacy of Caphosol is not known as an active treatment for patients with solid tumors suffering from mucositis induced by chemotherapy. Certainly, the incidence of oral mucositis is comparatively lower in patients with solid tumors treated with chemotherapy. However, mucositis will be seen more frequently in oncology patients with solid tumors as we will be increasingly using targeted therapies. In these patients, instead of a preventive treatment, amelioration of mucositis symptoms through an active therapy appears more reasonable.

Our case series has a number of limitations. In addition to being a retrospective analysis, we only followed 21 patients and these were selected on the basis of being treated with Caphosol, through a compassionate supply from the Canadian distributor. The severity, duration and recovery of neutropenia that could have potentially affected the improvement in mucositis were not recorded. The grade of mucositis were recorded by the physicians using the same scale, however, individual variation is possible. There were no side effects recorded with the use of Caphosol but this information is self-reported and subject to the recall bias.

As we have observed in our case series, Caphosol may be a potentially effective therapy in patients with solid tumors suffering from chemotherapy-induced mucositis. Despite the limitations listed for our case-series, we are encouraged by majority of patients responding so well to Caphosol. Since there has been no published trial or case-series evaluating the role of Caphosol as an active treatment for mucositis, this case series may be considered to provide some basis to conduct a larger randomized study in future.
